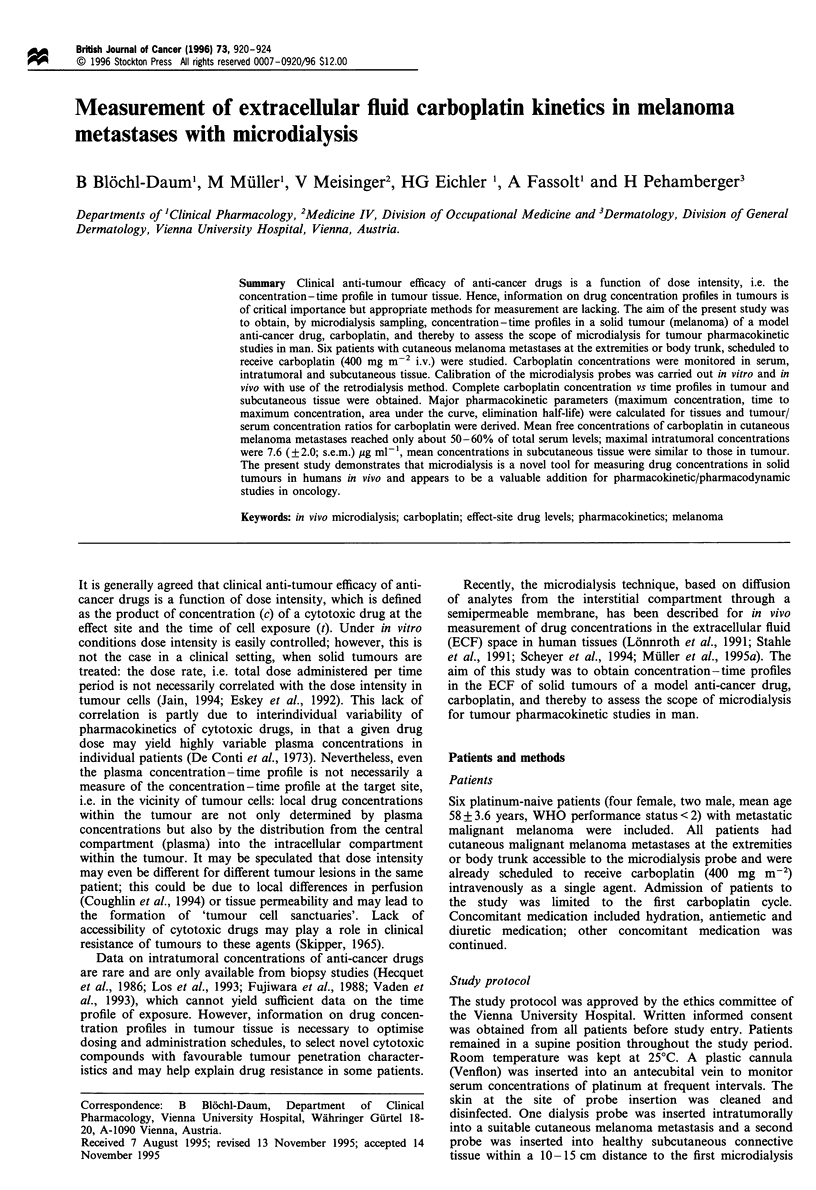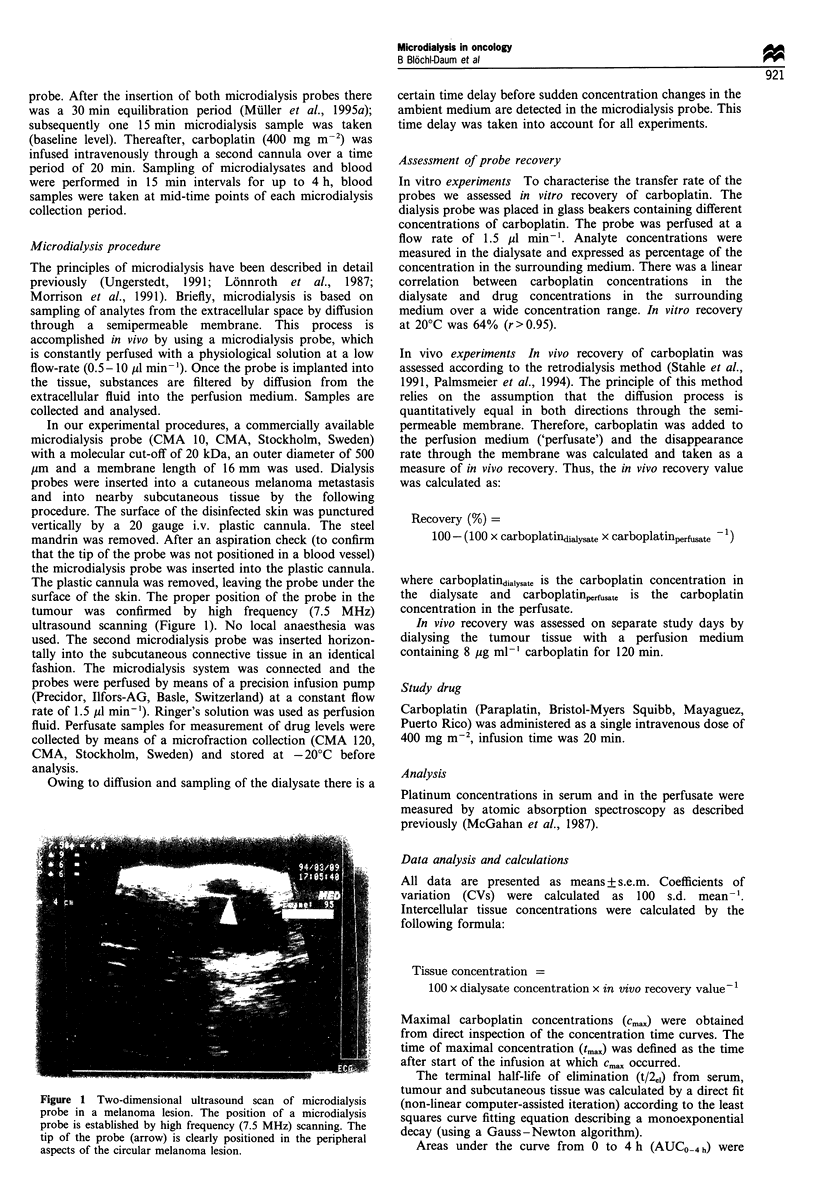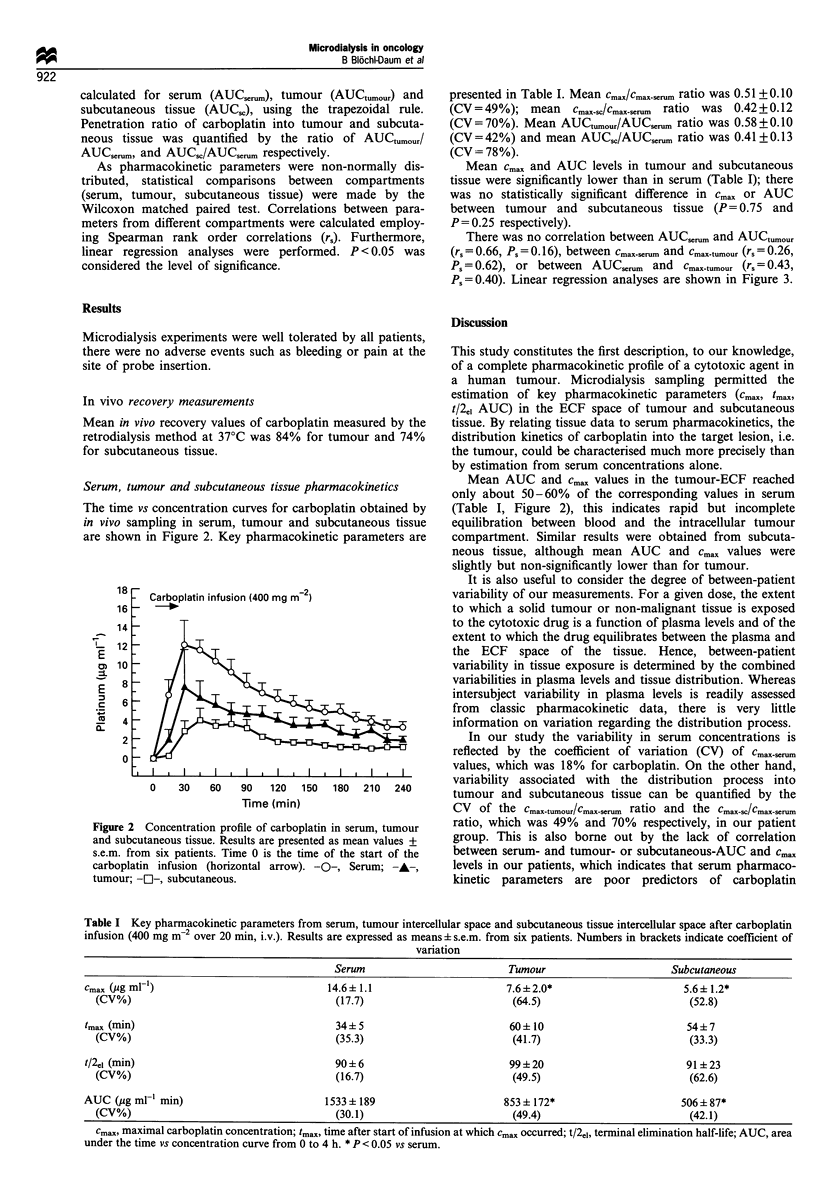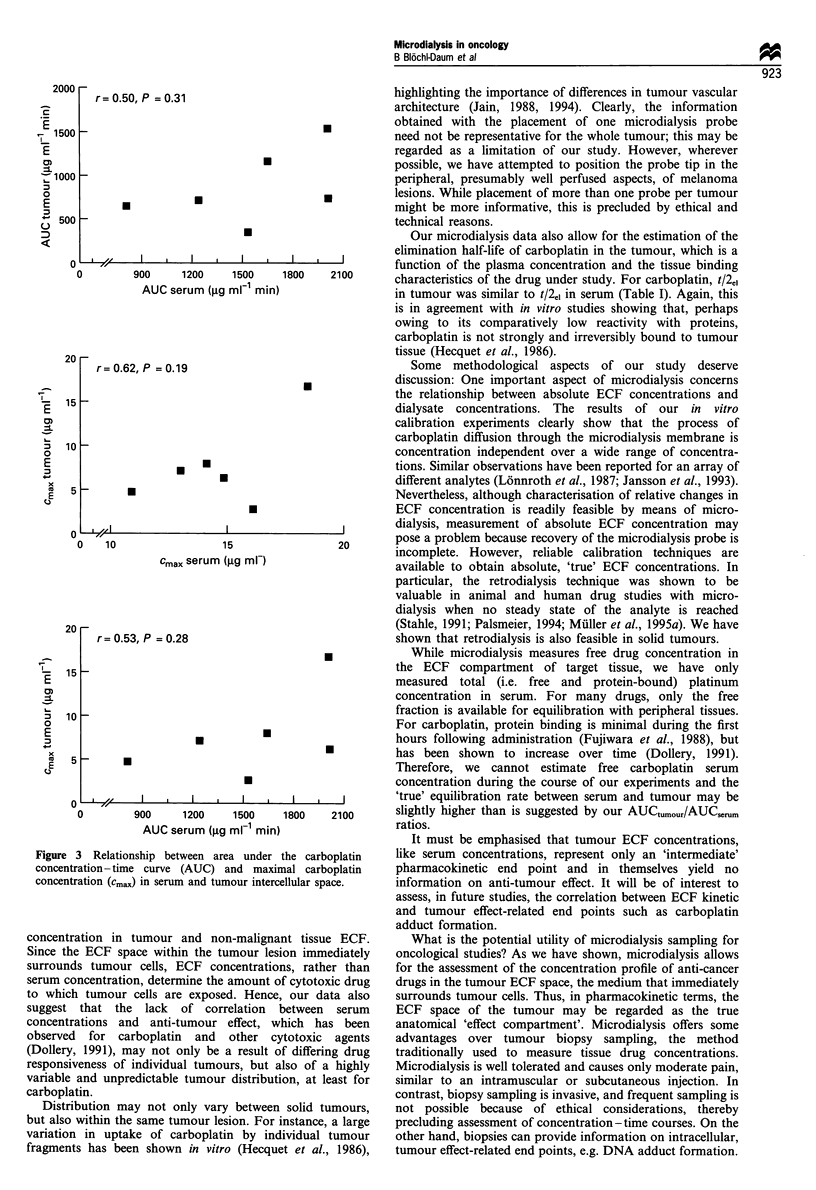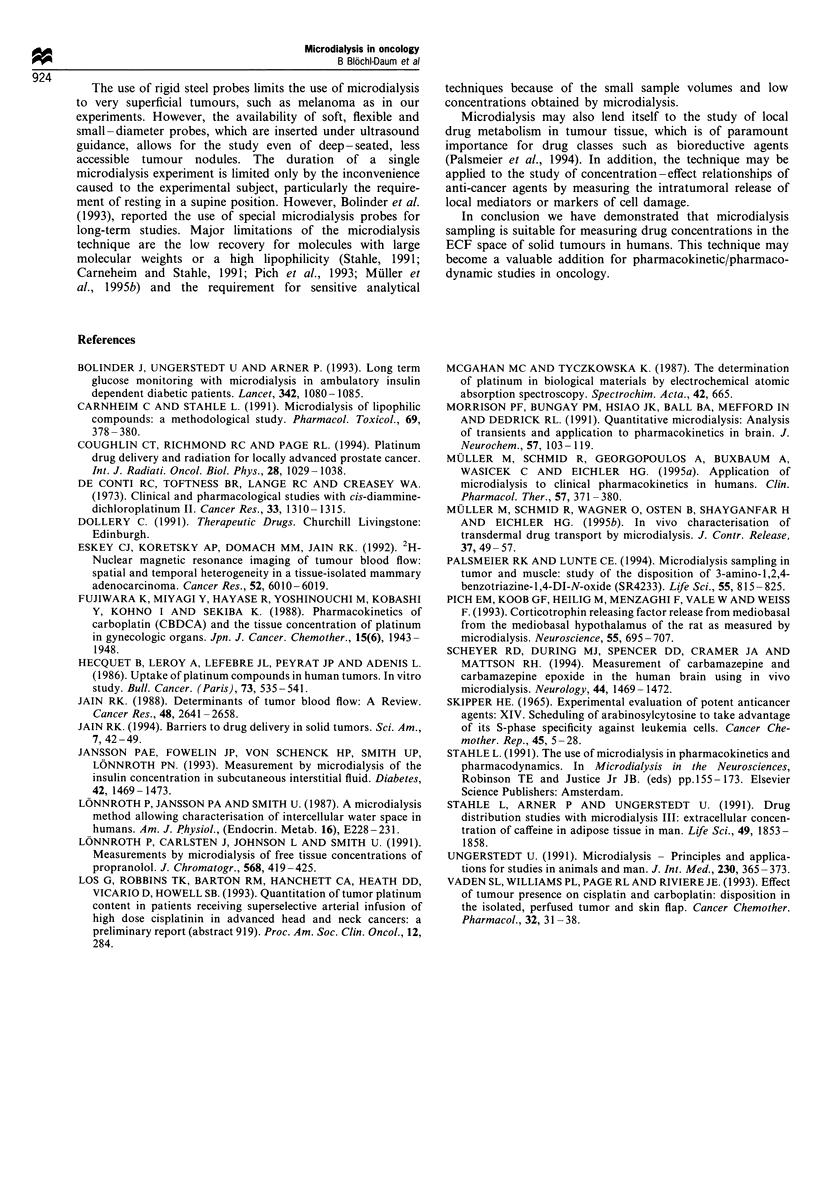# Measurement of extracellular fluid carboplatin kinetics in melanoma metastases with microdialysis.

**DOI:** 10.1038/bjc.1996.164

**Published:** 1996-04

**Authors:** B. Blöchl-Daum, M. Müller, V. Meisinger, H. G. Eichler, A. Fassolt, H. Pehamberger

**Affiliations:** Department of Clincal Pharmacology, Vienna University Hospital, Austria.

## Abstract

**Images:**


British Journal of Cancer (1996) 73, 920-924
© 1996 Stockton Press All rights reserved 0007-0920/96 $12.00

### Measurement of extracellular fluid carboplatin kinetics in melanoma metastases with microdialysis

B Blöchl-Daum[1], M Müller[1], V Meisinger[2], HG Eichler [1], A Fassolt[1] and H Pehamberger[3]

*Departments of [1]Clinical Pharmacology, [2]Medicine IV, Division of Occupational Medicine and [3]Dermatology, Division of General Dermatology, Vienna University Hospital, Vienna, Austria.*

**Summary** Clinical anti-tumour efficacy of anti-cancer drugs is a function of dose intensity, i.e. the concentration–time profile in tumour tissue. Hence, information on drug concentration profiles in tumours is of critical importance but appropriate methods for measurement are lacking. The aim of the present study was to obtain, by microdialysis sampling, concentration–time profiles in a solid tumour (melanoma) of a model anti-cancer drug, carboplatin, and thereby to assess the scope of microdialysis for tumour pharmacokinetic studies in man. Six patients with cutaneous melanoma metastases at the extremities or body trunk, scheduled to receive carboplatin (400 mg $m^{-2}$ i.v.) were studied. Carboplatin concentrations were monitored in serum, intratumoral and subcutaneous tissue. Calibration of the microdialysis probes was carried out *in vitro* and *in vivo* with use of the retrodialysis method. Complete carboplatin concentration *vs* time profiles in tumour and subcutaneous tissue were obtained. Major pharmacokinetic parameters (maximum concentration, time to maximum concentration, area under the curve, elimination half-life) were calculated for tissues and tumour/serum concentration ratios for carboplatin were derived. Mean free concentrations of carboplatin in cutaneous melanoma metastases reached only about 50–60% of total serum levels; maximal intratumoral concentrations were 7.6 ($\pm$2.0; s.e.m.) $\mu g\ ml^{-1}$, mean concentrations in subcutaneous tissue were similar to those in tumour. The present study demonstrates that microdialysis is a novel tool for measuring drug concentrations in solid tumours in humans *in vivo* and appears to be a valuable addition for pharmacokinetic/pharmacodynamic studies in oncology.

**Keywords:** *in vivo* microdialysis; carboplatin; effect-site drug levels; pharmacokinetics; melanoma

It is generally agreed that clinical anti-tumour efficacy of anti-cancer drugs is a function of dose intensity, which is defined as the product of concentration (*c*) of a cytotoxic drug at the effect site and the time of cell exposure (*t*). Under *in vitro* conditions dose intensity is easily controlled; however, this is not the case in a clinical setting, when solid tumours are treated: the dose rate, i.e. total dose administered per time period is not necessarily correlated with the dose intensity in tumour cells (Jain, 1994; Eskey *et al.*, 1992). This lack of correlation is partly due to interindividual variability of pharmacokinetics of cytotoxic drugs, in that a given drug dose may yield highly variable plasma concentrations in individual patients (De Conti *et al.*, 1973). Nevertheless, even the plasma concentration–time profile is not necessarily a measure of the concentration–time profile at the target site, i.e. in the vicinity of tumour cells: local drug concentrations within the tumour are not only determined by plasma concentrations but also by the distribution from the central compartment (plasma) into the intracellular compartment within the tumour. It may be speculated that dose intensity may even be different for different tumour lesions in the same patient; this could be due to local differences in perfusion (Coughlin *et al.*, 1994) or tissue permeability and may lead to the formation of 'tumour cell sanctuaries'. Lack of accessibility of cytotoxic drugs may play a role in clinical resistance of tumours to these agents (Skipper, 1965).

Data on intratumoral concentrations of anti-cancer drugs are rare and are only available from biopsy studies (Hecquet *et al.*, 1986; Los *et al.*, 1993; Fujiwara *et al.*, 1988; Vaden *et al.*, 1993), which cannot yield sufficient data on the time profile of exposure. However, information on drug concentration profiles in tumour tissue is necessary to optimise dosing and administration schedules, to select novel cytotoxic compounds with favourable tumour penetration characteristics and may help explain drug resistance in some patients.

Correspondence: B Blöchl-Daum, Department of Clinical Pharmacology, Vienna University Hospital, Währinger Gürtel 18-20, A-1090 Vienna, Austria.
Received 7 August 1995; revised 13 November 1995; accepted 14 November 1995

Recently, the microdialysis technique, based on diffusion of analytes from the interstitial compartment through a semipermeable membrane, has been described for *in vivo* measurement of drug concentrations in the extracellular fluid (ECF) space in human tissues (Lönnroth *et al.*, 1991; Stahle *et al.*, 1991; Scheyer *et al.*, 1994; Müller *et al.*, 1995*a*). The aim of this study was to obtain concentration–time profiles in the ECF of solid tumours of a model anti-cancer drug, carboplatin, and thereby to assess the scope of microdialysis for tumour pharmacokinetic studies in man.

### Patients and methods

### *Patients*

Six platinum-naive patients (four female, two male, mean age 58$\pm$3.6 years, WHO performance status < 2) with metastatic malignant melanoma were included. All patients had cutaneous malignant melanoma metastases at the extremities or body trunk accessible to the microdialysis probe and were already scheduled to receive carboplatin (400 mg $m^{-2}$) intravenously as a single agent. Admission of patients to the study was limited to the first carboplatin cycle. Concomitant medication included hydration, antiemetic and diuretic medication; other concomitant medication was continued.

### *Study protocol*

The study protocol was approved by the ethics committee of the Vienna University Hospital. Written informed consent was obtained from all patients before study entry. Patients remained in a supine position throughout the study period. Room temperature was kept at 25°C. A plastic cannula (Venflon) was inserted into an antecubital vein to monitor serum concentrations of platinum at frequent intervals. The skin at the site of probe insertion was cleaned and disinfected. One dialysis probe was inserted intratumorally into a suitable cutaneous melanoma metastasis and a second probe was inserted into healthy subcutaneous connective tissue within a 10–15 cm distance to the first microdialysis probe. After the insertion of both microdialysis probes there was a 30 min equilibration period (Müller *et al.*, 1995*a*); subsequently one 15 min microdialysis sample was taken (baseline level). Thereafter, carboplatin (400 mg $m^{-2}$) was infused intravenously through a second cannula over a time period of 20 min. Sampling of microdialysates and blood were performed in 15 min intervals for up to 4 h, blood samples were taken at mid-time points of each microdialysis collection period.

### *Microdialysis procedure*

The principles of microdialysis have been described in detail previously (Ungerstedt, 1991; Lönnroth *et al.*, 1987; Morrison *et al.*, 1991). Briefly, microdialysis is based on sampling of analytes from the extracellular space by diffusion through a semipermeable membrane. This process is accomplished *in vivo* by using a microdialysis probe, which is constantly perfused with a physiological solution at a low flow-rate (0.5 – 10 $\mu$l $min^{-1}$). Once the probe is implanted into the tissue, substances are filtered by diffusion from the extracellular fluid into the perfusion medium. Samples are collected and analysed.

In our experimental procedures, a commercially available microdialysis probe (CMA 10, CMA, Stockholm, Sweden) with a molecular cut-off of 20 kDa, an outer diameter of 500 $\mu$m and a membrane length of 16 mm was used. Dialysis probes were inserted into a cutaneous melanoma metastasis and into nearby subcutaneous tissue by the following procedure. The surface of the disinfected skin was punctured vertically by a 20 gauge i.v. plastic cannula. The steel mandrin was removed. After an aspiration check (to confirm that the tip of the probe was not positioned in a blood vessel) the microdialysis probe was inserted into the plastic cannula. The plastic cannula was removed, leaving the probe under the surface of the skin. The proper position of the probe in the tumour was confirmed by high frequency (7.5 MHz) ultrasound scanning (Figure 1). No local anaesthesia was used. The second microdialysis probe was inserted horizontally into the subcutaneous connective tissue in an identical fashion. The microdialysis system was connected and the probes were perfused by means of a precision infusion pump (Precidor, Ilfors-AG, Basle, Switzerland) at a constant flow rate of 1.5 $\mu$l $min^{-1}$). Ringer's solution was used as perfusion fluid. Perfusate samples for measurement of drug levels were collected by means of a microfraction collection (CMA 120, CMA, Stockholm, Sweden) and stored at −20°C before analysis.

Owing to diffusion and sampling of the dialysate there is a certain time delay before sudden concentration changes in the ambient medium are detected in the microdialysis probe. This time delay was taken into account for all experiments.

**Figure 1** Two-dimensional ultrasound scan of microdialysis probe in a melanoma lesion. The position of a microdialysis probe is established by high frequency (7.5 MHz) scanning. The tip of the probe (arrow) is clearly positioned in the peripheral aspects of the circular melanoma lesion.

### *Assessment of probe recovery*

In vitro *experiments* To characterise the transfer rate of the probes we assessed *in vitro* recovery of carboplatin. The dialysis probe was placed in glass beakers containing different concentrations of carboplatin. The probe was perfused at a flow rate of 1.5 $\mu$l $min^{-1}$. Analyte concentrations were measured in the dialysate and expressed as percentage of the concentration in the surrounding medium. There was a linear correlation between carboplatin concentrations in the dialysate and drug concentrations in the surrounding medium over a wide concentration range. *In vitro* recovery at 20°C was 64% ($r > 0.95$).

In vivo *experiments* *In vivo* recovery of carboplatin was assessed according to the retrodialysis method (Stahle *et al.*, 1991, Palmsmeier *et al.*, 1994). The principle of this method relies on the assumption that the diffusion process is quantitatively equal in both directions through the semipermeable membrane. Therefore, carboplatin was added to the perfusion medium ('perfusate') and the disappearance rate through the membrane was calculated and taken as a measure of *in vivo* recovery. Thus, the *in vivo* recovery value was calculated as:

$$\text{Recovery (\%)} = 100 - (100 \times \text{carboplatin}_{\text{dialysate}} \times \text{carboplatin}_{\text{perfusate}}{}^{-1})$$

where $\text{carboplatin}_{\text{dialysate}}$ is the carboplatin concentration in the dialysate and $\text{carboplatin}_{\text{perfusate}}$ is the carboplatin concentration in the perfusate.

*In vivo* recovery was assessed on separate study days by dialysing the tumour tissue with a perfusion medium containing 8 $\mu$g $ml^{-1}$ carboplatin for 120 min.

### *Study drug*

Carboplatin (Paraplatin, Bristol-Myers Squibb, Mayaguez, Puerto Rico) was administered as a single intravenous dose of 400 mg $m^{-2}$, infusion time was 20 min.

### *Analysis*

Platinum concentrations in serum and in the perfusate were measured by atomic absorption spectroscopy as described previously (McGahan *et al.*, 1987).

### *Data analysis and calculations*

All data are presented as means ± s.e.m. Coefficients of variation (CVs) were calculated as 100 s.d. $mean^{-1}$. Intercellular tissue concentrations were calculated by the following formula:

$$\text{Tissue concentration} = 100 \times \text{dialysate concentration} \times \textit{in vivo}\ \text{recovery value}^{-1}$$

Maximal carboplatin concentrations ($c_{max}$) were obtained from direct inspection of the concentration time curves. The time of maximal concentration ($t_{max}$) was defined as the time after start of the infusion at which $c_{max}$ occurred.

The terminal half-life of elimination ($t/2_{el}$) from serum, tumour and subcutaneous tissue was calculated by a direct fit (non-linear computer-assisted iteration) according to the least squares curve fitting equation describing a monoexponential decay (using a Gauss – Newton algorithm).

Areas under the curve from 0 to 4 h ($AUC_{0-4\,h}$) were calculated for serum ($AUC_{serum}$), tumour ($AUC_{tumour}$) and subcutaneous tissue ($AUC_{sc}$), using the trapezoidal rule. Penetration ratio of carboplatin into tumour and subcutaneous tissue was quantified by the ratio of $AUC_{tumour}/AUC_{serum}$, and $AUC_{sc}/AUC_{serum}$ respectively.

As pharmacokinetic parameters were non-normally distributed, statistical comparisons between compartments (serum, tumour, subcutaneous tissue) were made by the Wilcoxon matched paired test. Correlations between parameters from different compartments were calculated employing Spearman rank order correlations ($r_s$). Furthermore, linear regression analyses were performed. $P<0.05$ was considered the level of significance.

### Results

Microdialysis experiments were well tolerated by all patients, there were no adverse events such as bleeding or pain at the site of probe insertion.

### In vivo *recovery measurements*

Mean *in vivo* recovery values of carboplatin measured by the retrodialysis method at 37°C was 84% for tumour and 74% for subcutaneous tissue.

### *Serum, tumour and subcutaneous tissue pharmacokinetics*

The time *vs* concentration curves for carboplatin obtained by *in vivo* sampling in serum, tumour and subcutaneous tissue are shown in Figure 2. Key pharmacokinetic parameters are presented in Table I. Mean $c_{max}/c_{max\text{-}serum}$ ratio was $0.51 \pm 0.10$ (CV=49%); mean $c_{max\text{-}sc}/c_{max\text{-}serum}$ ratio was $0.42 \pm 0.12$ (CV=70%). Mean $AUC_{tumour}/AUC_{serum}$ ratio was $0.58 \pm 0.10$ (CV=42%) and mean $AUC_{sc}/AUC_{serum}$ ratio was $0.41 \pm 0.13$ (CV=78%).

**Figure 2** Concentration profile of carboplatin in serum, tumour and subcutaneous tissue. Results are presented as mean values ± s.e.m. from six patients. Time 0 is the time of the start of the carboplatin infusion (horizontal arrow). –○–, Serum; –▲–, tumour; –□–, subcutaneous.

Mean $c_{max}$ and AUC levels in tumour and subcutaneous tissue were significantly lower than in serum (Table I); there was no statistically significant difference in $c_{max}$ or AUC between tumour and subcutaneous tissue ($P=0.75$ and $P=0.25$ respectively).

There was no correlation between $AUC_{serum}$ and $AUC_{tumour}$ ($r_s=0.66$, $P_s=0.16$), between $c_{max\text{-}serum}$ and $c_{max\text{-}tumour}$ ($r_s=0.26$, $P_s=0.62$), or between $AUC_{serum}$ and $c_{max\text{-}tumour}$ ($r_s=0.43$, $P_s=0.40$). Linear regression analyses are shown in Figure 3.

### Discussion

This study constitutes the first description, to our knowledge, of a complete pharmacokinetic profile of a cytotoxic agent in a human tumour. Microdialysis sampling permitted the estimation of key pharmacokinetic parameters ($c_{max}$, $t_{max}$, $t/2_{el}$ AUC) in the ECF space of tumour and subcutaneous tissue. By relating tissue data to serum pharmacokinetics, the distribution kinetics of carboplatin into the target lesion, i.e. the tumour, could be characterised much more precisely than by estimation from serum concentrations alone.

Mean AUC and $c_{max}$ values in the tumour-ECF reached only about 50–60% of the corresponding values in serum (Table I, Figure 2), this indicates rapid but incomplete equilibration between blood and the intracellular tumour compartment. Similar results were obtained from subcutaneous tissue, although mean AUC and $c_{max}$ values were slightly but non-significantly lower than for tumour.

It is also useful to consider the degree of between-patient variability of our measurements. For a given dose, the extent to which a solid tumour or non-malignant tissue is exposed to the cytotoxic drug is a function of plasma levels and of the extent to which the drug equilibrates between the plasma and the ECF space of the tissue. Hence, between-patient variability in tissue exposure is determined by the combined variabilities in plasma levels and tissue distribution. Whereas intersubject variability in plasma levels is readily assessed from classic pharmacokinetic data, there is very little information on variation regarding the distribution process.

In our study the variability in serum concentrations is reflected by the coefficient of variation (CV) of $c_{max\text{-}serum}$ values, which was 18% for carboplatin. On the other hand, variability associated with the distribution process into tumour and subcutaneous tissue can be quantified by the CV of the $c_{max\text{-}tumour}/c_{max\text{-}serum}$ ratio and the $c_{max\text{-}sc}/c_{max\text{-}serum}$ ratio, which was 49% and 70% respectively, in our patient group. This is also borne out by the lack of correlation between serum- and tumour- or subcutaneous-AUC and $c_{max}$ levels in our patients, which indicates that serum pharmacokinetic parameters are poor predictors of carboplatin

**Table I** Key pharmacokinetic parameters from serum, tumour intercellular space and subcutaneous tissue intercellular space after carboplatin infusion (400 mg m$^{-2}$ over 20 min, i.v.). Results are expressed as means ± s.e.m. from six patients. Numbers in brackets indicate coefficient of variation

| | *Serum* | *Tumour* | *Subcutaneous* |
|---|---|---|---|
| $c_{max}$ (µg ml$^{-1}$) | 14.6 ± 1.1 | 7.6 ± 2.0* | 5.6 ± 1.2* |
| (CV%) | (17.7) | (64.5) | (52.8) |
| $t_{max}$ (min) | 34 ± 5 | 60 ± 10 | 54 ± 7 |
| (CV%) | (35.3) | (41.7) | (33.3) |
| $t/2_{el}$ (min) | 90 ± 6 | 99 ± 20 | 91 ± 23 |
| (CV%) | (16.7) | (49.5) | (62.6) |
| AUC (µg ml$^{-1}$ min) | 1533 ± 189 | 853 ± 172* | 506 ± 87* |
| (CV%) | (30.1) | (49.4) | (42.1) |

$c_{max}$, maximal carboplatin concentration; $t_{max}$, time after start of infusion at which $c_{max}$ occurred; $t/2_{el}$, terminal elimination half-life; AUC, area under the time *vs* concentration curve from 0 to 4 h. * $P<0.05$ *vs* serum.

**Figure 3** Relationship between area under the carboplatin concentration–time curve (AUC) and maximal carboplatin concentration ($c_{max}$) in serum and tumour intercellular space.

concentration in tumour and non-malignant tissue ECF. Since the ECF space within the tumour lesion immediately surrounds tumour cells, ECF concentrations, rather than serum concentration, determine the amount of cytotoxic drug to which tumour cells are exposed. Hence, our data also suggest that the lack of correlation between serum concentrations and anti-tumour effect, which has been observed for carboplatin and other cytotoxic agents (Dollery, 1991), may not only be a result of differing drug responsiveness of individual tumours, but also of a highly variable and unpredictable tumour distribution, at least for carboplatin.

Distribution may not only vary between solid tumours, but also within the same tumour lesion. For instance, a large variation in uptake of carboplatin by individual tumour fragments has been shown *in vitro* (Hecquet *et al.*, 1986), highlighting the importance of differences in tumour vascular architecture (Jain, 1988, 1994). Clearly, the information obtained with the placement of one microdialysis probe need not be representative for the whole tumour; this may be regarded as a limitation of our study. However, wherever possible, we have attempted to position the probe tip in the peripheral, presumably well perfused aspects, of melanoma lesions. While placement of more than one probe per tumour might be more informative, this is precluded by ethical and technical reasons.

Our microdialysis data also allow for the estimation of the elimination half-life of carboplatin in the tumour, which is a function of the plasma concentration and the tissue binding characteristics of the drug under study. For carboplatin, $t/2_{el}$ in tumour was similar to $t/2_{el}$ in serum (Table I). Again, this is in agreement with *in vitro* studies showing that, perhaps owing to its comparatively low reactivity with proteins, carboplatin is not strongly and irreversibly bound to tumour tissue (Hecquet *et al.*, 1986).

Some methodological aspects of our study deserve discussion: One important aspect of microdialysis concerns the relationship between absolute ECF concentrations and dialysate concentrations. The results of our *in vitro* calibration experiments clearly show that the process of carboplatin diffusion through the microdialysis membrane is concentration independent over a wide range of concentrations. Similar observations have been reported for an array of different analytes (Lönnroth *et al.*, 1987; Jansson *et al.*, 1993). Nevertheless, although characterisation of relative changes in ECF concentration is readily feasible by means of microdialysis, measurement of absolute ECF concentration may pose a problem because recovery of the microdialysis probe is incomplete. However, reliable calibration techniques are available to obtain absolute, 'true' ECF concentrations. In particular, the retrodialysis technique was shown to be valuable in animal and human drug studies with microdialysis when no steady state of the analyte is reached (Stahle, 1991; Palsmeier, 1994; Müller *et al.*, 1995*a*). We have shown that retrodialysis is also feasible in solid tumours.

While microdialysis measures free drug concentration in the ECF compartment of target tissue, we have only measured total (i.e. free and protein-bound) platinum concentration in serum. For many drugs, only the free fraction is available for equilibration with peripheral tissues. For carboplatin, protein binding is minimal during the first hours following administration (Fujiwara *et al.*, 1988), but has been shown to increase over time (Dollery, 1991). Therefore, we cannot estimate free carboplatin serum concentration during the course of our experiments and the 'true' equilibration rate between serum and tumour may be slightly higher than is suggested by our $AUC_{tumour}/AUC_{serum}$ ratios.

It must be emphasised that tumour ECF concentrations, like serum concentrations, represent only an 'intermediate' pharmacokinetic end point and in themselves yield no information on anti-tumour effect. It will be of interest to assess, in future studies, the correlation between ECF kinetic and tumour effect-related end points such as carboplatin adduct formation.

What is the potential utility of microdialysis sampling for oncological studies? As we have shown, microdialysis allows for the assessment of the concentration profile of anti-cancer drugs in the tumour ECF space, the medium that immediately surrounds tumour cells. Thus, in pharmacokinetic terms, the ECF space of the tumour may be regarded as the true anatomical 'effect compartment'. Microdialysis offers some advantages over tumour biopsy sampling, the method traditionally used to measure tissue drug concentrations. Microdialysis is well tolerated and causes only moderate pain, similar to an intramuscular or subcutaneous injection. In contrast, biopsy sampling is invasive, and frequent sampling is not possible because of ethical considerations, thereby precluding assessment of concentration–time courses. On the other hand, biopsies can provide information on intracellular, tumour effect-related end points, e.g. DNA adduct formation.

The use of rigid steel probes limits the use of microdialysis to very superficial tumours, such as melanoma as in our experiments. However, the availability of soft, flexible and small-diameter probes, which are inserted under ultrasound guidance, allows for the study even of deep-seated, less accessible tumour nodules. The duration of a single microdialysis experiment is limited only by the inconvenience caused to the experimental subject, particularly the requirement of resting in a supine position. However, Bolinder *et al.* (1993), reported the use of special microdialysis probes for long-term studies. Major limitations of the microdialysis technique are the low recovery for molecules with large molecular weights or a high lipophilicity (Stahle, 1991; Carneheim and Stahle, 1991; Pich *et al.*, 1993; Müller *et al.*, 1995*b*) and the requirement for sensitive analytical techniques because of the small sample volumes and low concentrations obtained by microdialysis.

Microdialysis may also lend itself to the study of local drug metabolism in tumour tissue, which is of paramount importance for drug classes such as bioreductive agents (Palsmeier *et al.*, 1994). In addition, the technique may be applied to the study of concentration-effect relationships of anti-cancer agents by measuring the intratumoral release of local mediators or markers of cell damage.

In conclusion we have demonstrated that microdialysis sampling is suitable for measuring drug concentrations in the ECF space of solid tumours in humans. This technique may become a valuable addition for pharmacokinetic/pharmacodynamic studies in oncology.

### References

BOLINDER J, UNGERSTEDT U AND ARNER P. (1993). Long term glucose monitoring with microdialysis in ambulatory insulin dependent diabetic patients. *Lancet*, **342**, 1080-1085.

CARNHEIM C AND STAHLE L. (1991). Microdialysis of lipophilic compounds: a methodological study. *Pharmacol. Toxicol.*, **69**, 378-380.

COUGHLIN CT, RICHMOND RC AND PAGE RL. (1994). Platinum drug delivery and radiation for locally advanced prostate cancer. *Int. J. Radiati. Oncol. Biol. Phys.*, **28**, 1029-1038.

DE CONTI RC, TOFTNESS BR, LANGE RC AND CREASEY WA. (1973). Clinical and pharmacological studies with *cis*-diamminedichloroplatinum II. *Cancer Res.*, **33**, 1310-1315.

DOLLERY C. (1991). *Therapeutic Drugs*. Churchill Livingstone: Edinburgh.

ESKEY CJ, KORETSKY AP, DOMACH MM, JAIN RK. (1992). $^2$H-Nuclear magnetic resonance imaging of tumour blood flow: spatial and temporal heterogeneity in a tissue-isolated mammary adenocarcinoma. *Cancer Res.*, **52**, 6010-6019.

FUJIWARA K, MIYAGI Y, HAYASE R, YOSHINOUCHI M, KOBASHI Y, KOHNO I AND SEKIBA K. (1988). Pharmacokinetics of carboplatin (CBDCA) and the tissue concentration of platinum in gynecologic organs. *Jpn. J. Cancer. Chemother.*, **15(6)**, 1943-1948.

HECQUET B, LEROY A, LEFEBRE JL, PEYRAT JP AND ADENIS L. (1986). Uptake of platinum compounds in human tumors. In vitro study. *Bull. Cancer. (Paris)*, **73**, 535-541.

JAIN RK. (1988). Determinants of tumor blood flow: A Review. *Cancer Res.*, **48**, 2641-2658.

JAIN RK. (1994). Barriers to drug delivery in solid tumors. *Sci. Am.*, **7**, 42-49.

JANSSON PAE, FOWELIN JP, VON SCHENCK HP, SMITH UP, LÖNNROTH PN. (1993). Measurement by microdialysis of the insulin concentration in subcutaneous interstitial fluid. *Diabetes*, **42**, 1469-1473.

LÖNNROTH P, JANSSON PA AND SMITH U. (1987). A microdialysis method allowing characterisation of intercellular water space in humans. *Am. J. Physiol.*, (Endocrin. Metab. **16**), E228-231.

LÖNNROTH P, CARLSTEN J, JOHNSON L AND SMITH U. (1991). Measurements by microdialysis of free tissue concentrations of propranolol. *J. Chromatogr.*, **568**, 419-425.

LOS G, ROBBINS TK, BARTON RM, HANCHETT CA, HEATH DD, VICARIO D, HOWELL SB. (1993). Quantitation of tumor platinum content in patients receiving superselective arterial infusion of high dose cisplatinin in advanced head and neck cancers: a preliminary report (abstract 919). *Proc. Am. Soc. Clin. Oncol.*, **12**, 284.

MCGAHAN MC AND TYCZKOWSKA K. (1987). The determination of platinum in biological materials by electrochemical atomic absorption spectroscopy. *Spectrochim. Acta.*, **42**, 665.

MORRISON PF, BUNGAY PM, HSIAO JK, BALL BA, MEFFORD IN AND DEDRICK RL. (1991). Quantitative microdialysis: Analysis of transients and application to pharmacokinetics in brain. *J. Neurochem.*, **57**, 103-119.

MÜLLER M, SCHMID R, GEORGOPOULOS A, BUXBAUM A, WASICEK C AND EICHLER HG. (1995*a*). Application of microdialysis to clinical pharmacokinetics in humans. *Clin. Pharmacol. Ther.*, **57**, 371-380.

MÜLLER M, SCHMID R, WAGNER O, OSTEN B, SHAYGANFAR H AND EICHLER HG. (1995*b*). In vivo characterisation of transdermal drug transport by microdialysis. *J. Contr. Release*, **37**, 49-57.

PALSMEIER RK AND LUNTE CE. (1994). Microdialysis sampling in tumor and muscle: study of the disposition of 3-amino-1,2,4-benzotriazine-1,4-DI-*N*-oxide (SR4233). *Life Sci.*, **55**, 815-825.

PICH EM, KOOB GF, HEILIG M, MENZAGHI F, VALE W AND WEISS F. (1993). Corticotrophin releasing factor release from mediobasal from the mediobasal hypothalamus of the rat as measured by microdialysis. *Neuroscience*, **55**, 695-707.

SCHEYER RD, DURING MJ, SPENCER DD, CRAMER JA AND MATTSON RH. (1994). Measurement of carbamazepine and carbamazepine epoxide in the human brain using in vivo microdialysis. *Neurology*, **44**, 1469-1472.

SKIPPER HE. (1965). Experimental evaluation of potent anticancer agents: XIV. Scheduling of arabinosylcytosine to take advantage of its S-phase specificity against leukemia cells. *Cancer Chemother. Rep.*, **45**, 5-28.

STAHLE L. (1991). The use of microdialysis in pharmacokinetics and pharmacodynamics. In *Microdialysis in the Neurosciences*, Robinson TE and Justice Jr JB. (eds) pp.155-173. Elsevier Science Publishers: Amsterdam.

STAHLE L, ARNER P AND UNGERSTEDT U. (1991). Drug distribution studies with microdialysis III: extracellular concentration of caffeine in adipose tissue in man. *Life Sci.*, **49**, 1853-1858.

UNGERSTEDT U. (1991). Microdialysis - Principles and applications for studies in animals and man. *J. Int. Med.*, **230**, 365-373.

VADEN SL, WILLIAMS PL, PAGE RL AND RIVIERE JE. (1993). Effect of tumour presence on cisplatin and carboplatin: disposition in the isolated, perfused tumor and skin flap. *Cancer Chemother. Pharmacol.*, **32**, 31-38.